# Comparison of clinical, immunological and virological markers in patients with HTLV-1 / HIV-1 co-infection with those with mono-infection

**DOI:** 10.1186/1742-4690-12-S1-P22

**Published:** 2015-08-28

**Authors:** D Dhasmana, A Puri, K Buell, G Taylor

**Affiliations:** 1 National Centre for Human Retrovirology, St Mary's Hospital, Paddington, London, UK

## 

This is a study of the HTLV-1/HIV-1 co-infected cohort attending the National Centre for Human Retrovirology in London. In a retrospective analysis, demographic, clinical, immunological and virological data was described. A subgroup of patients on combination antiretroviral therapy (cART) for more than twelve months with an undetectable HIV viral load was identified and compared to a matched HTLV-1 mono-infected group. In an ongoing prospective study, HIV-1 mono-infected patients are recruited and virological and immunological parameters compared to the HTLV/HIV co-infected and HTLV mono-infected groups. 29 co-infected patients had a median age of 47 years, 55% were male and over 80% identified with the Black – African/Caribbean ethnic group. The median time on cART was 6 months. CD4/CD4% cell counts were lower in stable co-infected patients (508 cells/mm3, p=0.2 / 27.9%, p<0.1) than HTLV mono-infected patients (941 cells/mm3/46.2%). There was a significant trend towards increased markers of T cell activation (TCA) in co-infected patients (Table 1), when compared to TCA markers in either mono-infected group. There were no statistically significant differences in pro-inflammatory cytokines between co-infected and mono-infected patients. However, there was a trend towards higher median interleukin-2 and gamma interferon concentrations in the co-infected group. Response to cART was not blunted in co-infection, and there was normalisation of CD4 counts over 12 months. In one patient absolute HTLV pVL (pro-viral load) increased 12 months after treatment with cART. The results demonstrate that there are differences between HTLV/HIV co-infected and mono-infected patients; notably increased immune activation, despite successful treatment with cART. It is hypothesised that co-infection creates an environmental milieu, reflected by increased immune TCA, that is associated with a higher prevalence of HTLV associated myelopathy that is observed in this group.

